# ProbSeq—A Fragmentation Model for Interpretation of Electrospray Tandem Mass Spectrometry Data

**DOI:** 10.1002/cfg.370

**Published:** 2004-02

**Authors:** John Skilling, Richard Denny, Keith Richardson, Phillip Young, Thérèse McKenna, Iain Campuzano, Mark Ritchie

**Affiliations:** 1 Maximum Entropy Data Consultants Ltd Tresawsan Killaha East Kenmare Ireland; 2 Waters Corporation MS Technologies Centre Floats Road Wythenshawe Manchester M23 9LZ UK

## Abstract

We describe a probabilistic peptide fragmentation model for use in protein databank searching and *de novo* sequencing of electrospray tandem mass spectrometry data. A probabilistic framework for tuning of the model using a range of well-characterized samples are introduced. We present preliminary results of our tuning efforts.


**Comparative and Functional Genomics**
*Comp Funct Genom* 2004; **5**: 61–68.
Published online in Wiley InterScience (www.interscience.wiley.com). **DOI:** 10.1002/cfg.370

**Conference Paper**

### ProbSeq — a fragmentation model for interpretation of electrospray tandem mass spectrometry data

John Skilling[1], Richard Denny[2], Keith Richardson[2]*, Phillip Young[2], Thérèse McKenna[2], Iain Campuzano[2] and Mark Ritchie[2]
[1]*Maximum Entropy Data Consultants Ltd, Tresawsan, Killaha East, Kenmare, Ireland*
[2]*Waters Corporation MS Technologies Centre, Floats Road, Wythenshawe, Manchester M23 9LZ, UK*

**Correspondence to: Keith Richardson, Waters Corporation MS Technologies Centre, Floats Road, Wythenshawe, Manchester M23 9LZ, UK. E-mail: keith_richardson@waters.com*

Received: 31 October 2003
Revised: 18 November 2003
Accepted: 26 November 2003

### Abstract

**We describe a probabilistic peptide fragmentation model for use in protein databank searching and *de novo* sequencing of electrospray tandem mass spectrometry data. A probabilistic framework for tuning of the model using a range of well-characterized samples are introduced. We present preliminary results of our tuning efforts.**
**Copyright © 2004 John Wiley & Sons, Ltd.**

### Introduction

Sequencing of peptides from their collision-induced fragments is a well-established technique (Papayannopoulos, 1995). A biological sample, which may contain an unknown protein or a mixture of proteins, is subjected to an enzymatic digest (usually tryptic) resulting in a mixture of peptides. This mixture is analysed using liquid chromatography and electrospray quadrupole time-of-flight (Q-TOF) mass spectrometry.

A Q-TOF (Morris *et al.*, 1996) mass spectrometer consists of a quadrupole mass analyser, a collision cell filled with an inert gas, and a time-of-flight analyser. The quadrupole allows the mass:charge range of peptides which enter the collision cell to be selected accurately. The Q-TOF can operate in two modes. In wide-bandpass, low-energy mode, all peptides are allowed to pass into the collision cell and the voltage applied to the collision cell is low, allowing the peptides to pass through intact into the TOF stage. In narrow-bandpass, high-energy mode, the quadrupole is tuned to pass only a small mass:charge range around a single peptide. In this mode, the collision cell voltage is relatively high, causing the peptide to fragment as it is pulled through the gas. The final, TOF stage of the instrument allows the peptide ion or fragment ion masses to be determined to an accuracy of around five parts per million on a well-calibrated instrument. The decision to switch mode is made in real time using a number of possible strategies, and the collision energy can be adjusted to make useful fragmentation of each peptide more likely.

Peptides fragment in complex ways, and their fragments in turn have complex representations in mass spectra. Manual interpretation of these spectra is a skilled and laborious job, and a modern experiment can produce many hundreds. Around 1998 (see Skilling, 2000a, 2000b), we began to use the ProbSeq fragmentation model in combination with Markov chain methods to interpret this data. Commonly, we are required to answer two questions: ‘How much can we infer

Copyright © 2004 John Wiley & Sons, Ltd.

about the sequence of the peptide which gave rise to a single spectrum?' and 'Given many spectra, what can we infer about the mixture of proteins that may be present?'. To address these questions in a robust, probabilistic way, we appeal to Bayes' theorem:

$$\begin{aligned}\Pr(\text{Sequence} \mid \text{Spectrum}, \mathcal{I}) &= \frac{\text{Prior} \times \text{Likelihood}}{\text{Evidence}} \\ &= \frac{\Pr(\text{Sequence} \mid \mathcal{I})\ \Pr(\text{Spectrum} \mid \text{Sequence}, \mathcal{I})}{\Pr(\text{Spectrum} \mid \mathcal{I})}.\end{aligned} \tag{1}$$

The 'Likelihood' $\Pr(\text{Spectrum} \mid \text{Sequence}, \mathcal{I})$ on the RHS of the above equation encodes our incomplete knowledge about peptide fragmentation. It is the probability that the peptide under consideration could give rise to the observed spectrum. The 'Prior' $\Pr(\text{Sequence} \mid \mathcal{I})$ is a probability that we assign to each candidate sequence before we look at the data. At this level, the 'Evidence' is a normalization factor, but it can be used to objectively compare different likelihood functions and other background assumptions. $\mathcal{I}$ contains information about the context in which this calculation is being used, and it happens that it is different for each of the above questions.

Our likelihood function is called 'ProbSeq' and it is, in itself, a Bayesian calculation which involves a good deal of prior information about peptide fragmentation. The rest of this paper introduces a new framework which we can use to improve the model. We regard it to be a strength of the Bayesian approach that we can treat this as an inference problem at every level (Skilling, 1998).

### Materials and methods

We decided to base the tuning of the likelihood function on human assignments of sequences to spectra. The samples chosen consisted of tryptic digests of pyruvate kinase, glyceraldehyde 3-phosphate dehydrogenase, alcohol dehydrogenase and $\beta$-lactoglobulin. The solvent was 0.1% formic acid in acetonitrile:water, 1:1. The samples were infused directly into a nanolockspray source. The instrument used was a Waters Q-TOF ULTIMA™ operating in positive ion mode. The collision energy was 20–45 eV.

The acquired spectra were analysed manually to provide a list of verified sequences. The data was processed using the MaxEnt3™ deconvolution algorithm to remove isotope series and to resolve overlapping isotope clusters and multiple charge states. The data was split into two sets. The first, 'tuning', set was used to tune the fragmentation model, while the second, 'validation', set was reserved for assessment of the resulting parameters.

The chemical structure and fragmentation modes for a typical peptide are shown in Figure 1. The types of fragments that are observed depends on the collision energy used. The calculation of the likelihood is based on a probabilistic summation over all of the possible ways that a peptide could fragment and give rise to trial masses:

$$\begin{aligned}&\Pr(\text{Spectrum} \mid \text{Sequence}, \Psi, \mathcal{I}) \\ &= \sum_{\text{Frag}} \Pr(\text{Spectrum} \mid \text{Frag}) \\ &\quad \times \Pr(\text{Frag} \mid \text{Sequence}, \Psi, \mathcal{I})\end{aligned} \tag{2}$$

where $\Psi$ is a database containing probabilistic information about peptide fragmentation and 'Frag' is a particular fragmentation pattern. Experience tells us that some patterns of fragmentation are more likely than others, e.g. $y$ ion series are correlated: if $y_n$ is present, there are better than even odds that $y_{n+1}$ is also present. This is the information that is encoded in the last term in equation (2). For a peptide of length $n$ amino acids, the sum in equation (2) contains $2^n$ terms for $y$ ions alone and $2^{6n}$ terms if $a$,$b$,$c$,$x$,$y$,$z$-ions are all considered with extra factors for various losses. It would be impractical to do this summation explicitly for each trial sequence. Remarkably, using a Markov chain allows the summation to be performed in $O(n)$ steps. Considering only $y$ ions, the chain is structured as follows:

$$\begin{aligned}\Pr(y_1 \text{ on}) &= p_1 \\ \text{and } \Pr(y_r \text{ on}) &= p_r \Pr(y_{r-1} \text{ on}) \\ &\quad + q_r \Pr(y_{r-1} \text{ off}).\end{aligned} \tag{3}$$

As mentioned above, the probability $p$ that the $y$ series stays on is expected to be greater than 0.5, and the probability $q$ that it switches on is likely to

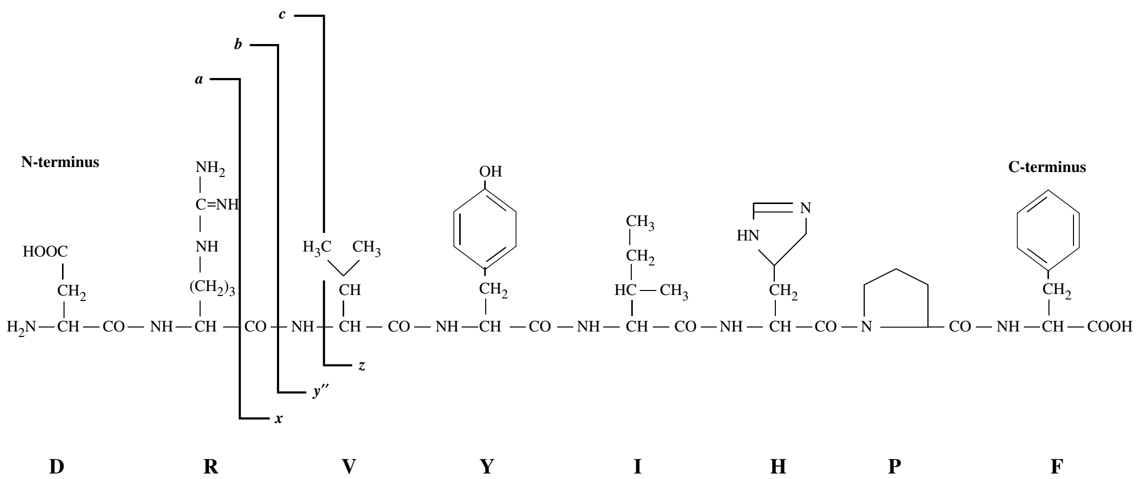

**Figure 1.** The chemical structure of a peptide. The boldface letters are one-letter amino acid codes. Also indicated are the three backbone fragmentation modes. At the collision energies used in this study, the *b*, *y*″ mode dominates. *a* and *z* ions are also commonly observed. Losses can also occur from terminal and side-chain groups be less than 0.5. It is also to be expected that the values of $p$ and $q$ at a particular point in the chain should depend on the amino acid sequence of the peptide under consideration. At each step along the chain, the probability that a $y$ ion will be observed is influenced by whether or not its predecessor was observed and we do as many summations as are locally available. When we reach the end of the chain, we have accumulated Pr(Spectrum | Sequence, $\Psi, \mathcal{I}$), with a computational cost of only $O(n)$.

As well as the Markov chain parameters which describe the expected appearance of series of $b$ and $y$ ions, each amino acid may undergo a number of losses, may exhibit a propensity for cleavage to occur on the C- or N-terminal side and may appear isolated from the rest of the sequence as an immonium ion. The maximum number of probabilities required to encode this description is seven per amino acid. This leads to our fragmentation model, $\Psi$, having over 100 tuneable parameters. Some examples are given in Table 1. Reasonable values for some of these can be arrived at by consulting the literature (Papayannopoulos, 1995), and others can be estimated by manually inspecting many fragmentation spectra.

We decided to treat the tuning of the ProbSeq parameters as another exercise in Bayesian inference. In order to do this we needed to define prior distributions for our parameters and a likelihood function for comparison against the data. Having a list of sequences validated against known spectra, the tuning likelihood becomes:

**Table 1.** Some examples of tuneable likelihood parameters. The first column contains probabilities that apply globally, while the second column contains probabilities that may be different for each amino acid

| Global | Amino Acid |
|---|---|
| Pr($y_{n+1}$ on \| $y_n$ on) | Pr(break left) |
| Pr($y_{n+1}$ off \| $y_n$ on) | Pr(break right) |
| Pr($y - H_2O$) | Pr(Immonium) |

$$\begin{aligned}&\text{Pr(Spectra} \mid \text{Sequences}, \Psi, \mathcal{I})\\ &= \prod_{\text{Spectra}} \text{Pr(Spectrum} \mid \text{Sequence}, \Psi, \mathcal{I}) \quad (4)\end{aligned}$$

We take the prior distribution for each of the probabilities in the ProbSeq model, $\Psi$, to be uniform on $(0, 1)$. We are left with the problem of how to explore the $>100$-dimensional parameter space. We validate the result by checking whether a chosen set of parameters, $\Psi$, does or does not enable a *de novo* sequence determination to recover the correct, known, sequence. This has the advantage of tuning our parameters directly upon our own implementation software.

*De novo* sequencing involves the exploration of a large space of peptide sequences that are consistent

Copyright © 2004 John Wiley & Sons, Ltd. 

with the intact mass of an unknown peptide. At least some of the candidate sequences must be compared with the associated fragmentation spectrum. The number of possible trial sequences grows exponentially with precursor mass with:

$$\log_2 n \approx \frac{M}{25} - 7 \tag{5}$$

where $n$ is the number of trial sequences and $M$ is the nominal mass of the precursor. The implementation used in the Waters proteomics product, ProteinLynx Global Server™, does not use an exhaustive search but simulates this by sampling from the space of possible peptide sequences through a terminated Markov Chain Monte Carlo (MCMC) algorithm. Initially, an ensemble of trial solutions is constructed by sampling from a prior distribution. The prior probability of a trial peptide is based on the natural abundances of its constituent amino acids and the preference for C-terminal residues, if appropriate, for a particular digestion reagent. In order to proceed, new trial sequences must be generated. We employ transition engines that change the state of an ensemble member, with a probability of transition, $T$, defined by:

$$\frac{T(i \to j)}{T(j \to i)} = \frac{\text{Prior}(j)}{\text{Prior}(i)} \tag{6}$$

where $i$ and $j$ represent two possible solutions in the space. Transitions of this type will eventually converge on occupancies of states which match the prior distribution. The various transition schemes employed by the *de novo* algorithm are outlined in Table 2. The data must be introduced via the likelihood function (ProbSeq in this case), as we wish to progress from the prior to the posterior. The transition probabilities are therefore combined with acceptance probabilities, $A$, where:

$$\frac{A(i \to j)T(i \to j)}{A(j \to i)T(j \to i)} = \frac{\text{Prior}(j)\text{Likelihood}(j)}{\text{Prior}(i)\text{Likelihood}(i)} = \frac{\text{Posterior}(j)}{\text{Posterior}(i)} \tag{7}$$

so that:

$$\frac{A(i \to j)}{A(j \to i)} = \frac{\text{Likelihood}(j)}{\text{Likelihood}(i)} \tag{8}$$

**Table 2.** The MCMC transitions for peptide sequencing: reversal of a contiguous subsequence with randomly chosen end-points, rotation of a contiguous subsequence with randomly chosen end-points, permutation of a contiguous subsequence with randomly chosen end-points, replacement of a contiguous subsequence with randomly chosen end-points, exchange of the C-terminus and N-terminus ends of two sequences to preserve nominal mass. The last transition is an example of a 'genetic algorithm'

| Type | Before | After |
|---|---|---|
| Reversal | XXXARQEIKXXX | XXXKIEQRAXXX |
| Rotation | XXXARQEIKXXX | XXXQEIKARXXX |
| Permutation | XXXARQEIKXXX | XXXIQRKAEXXX |
| Replacement | XXXNEQXXX | XXXEKGGXXX |
| Exchange | EKGG-DQCYKR, NEH-YKDQCR | NEH-DQCYKR, EKGG-YKDQCR |

This is the usual Metropolis–Hastings approach of detailed balancing (Metropolis *et al.*, 1953; Hastings, 1970). In order to improve convergence, the likelihood is introduced in a modified form, by raising it to some power $0 \leq \lambda \leq 1$. Here, $\lambda$ is analogous to an inverse temperature and by raising it slowly from 0 to 1 (i.e. by slow cooling), we implement 'simulated annealing' (Kirkpatrick *et al.*, 1983). We use simulated annealing for both *de novo* exploration and exploring the ProbSeq parameter space, $\Psi$ (see Table 3 for an outline of the commonalities).

The result of a *de novo* exploration is a number of candidate peptide sequences that may account for the fragmentation spectrum and precursor mass, accompanied by a posterior probability. In order to assess whether the results of tuning the ProbSeq likelihood parameters afford greater discrimination in favour of the correct sequences, we can perform *de novo* searches and inspect the ranks and posterior probabilities of the correct sequences. Indeed,

**Table 3.** Comparison between tuning and sequencing, which are both inference problems that can be explored using MCMC methods

| | Probseq tuning | *De novo* sequencing |
|---|---|---|
| Likelihood | Pr(Spectrum \| $\Psi$) | Pr(Spectrum \| Sequence) |
| Space | $[0, 1]^n$-unit hypercube | Discrete space of $\sim 10^{10}$ sequences |
| Prior | Uniform | Given by amino acid abundance |

Copyright © 2004 John Wiley & Sons, Ltd. 

we could have replaced equation (4) in favour of a likelihood for the tuning parameters which incorporated *de novo* searches in order to involve this discrimination directly in the tuning process. However, the exploration of the parameter space would have become prohibitively time-consuming.

### Results and Discussion

Although cysteine and tryptophan are under-represented in the tuning dataset, the probabilities for immonium production allow us to make general comparisons with observations described in the literature. Papayannopoulos (1995) indicates that arginine, lysine, leucine/isoleucine, cysteine, histidine, phenylalanine, tyrosine and tryptophan may give diagnostically important immonium ions (see Table 4). Our results indicate that arginine, leucine/isoleucine, cysteine, histidine, phenylalanine, tyrosine and tryptophan are likely to give stronger signals, although the strengths reported may be sensitive to the configuration of the instrument.

The results of performing *de novo* searches on the tuning and validation datasets are summarized in Tables 5 and 6. There is a noticeable improvement in the results of *de novo* sequencing in the tuning dataset: the correct sequence is ranked first on eight occasions with the tuned prior as opposed to six with the original prior out of a total of 39 sequences. With the tuning dataset, 17 correct sequences appeared in the top 10 with the tuned prior, against 10 for the original prior.

The improvement is less clear in the validation dataset but is still present, with 11 correct for the tuned prior and nine for the original prior out of 31 sequences; 17 correct sequences appeared in the top 10 for both the tuned and original priors.

The improvements gained by tuning the prior probabilities of the ProbSeq model are slight. This is to be expected as, when accurately mass-measured data are available, the information in the data dominates the prior. The quality of our results is not critically dependent on specific parameter settings, which is reassuring. We may have gained some improvement for marginal data but this remains to be seen, perhaps through tuning and validation with a more extensive library of data.

Overall, tuning has resulted in the softening of some probabilities, e.g. those for immonium

**Table 4.** Listed for each amino acid are occurrences in the validated sequences throughout the tuning dataset, occurrences in different spectra, mean probability of immonium ion production and its standard deviation as sampled from the posterior distribution during the MCMC exploration

| Amino acid | Occurrences | Spectra | Pr(Immonium) | Standard deviation |
|---|---|---|---|---|
| Alanine | 57 | 25 | 0.0 | 0.0 |
| Arginine* | 12 | 12 | 0.2418 | 0.0622 |
| Asparagine | 13 | 10 | 0.0334 | 0.0234 |
| Aspartic acid | 47 | 28 | 0.0071 | 0.0068 |
| Cysteine* | 1 | 1 | 0.2205 | 0.0695 |
| Glutamine | 8 | 7 | 0.0702 | 0.0514 |
| Glutamic acid | 47 | 22 | 0.1829 | 0.0375 |
| Glycine | 26 | 16 | 0.0 | 0.0 |
| Histidine* | 7 | 7 | 0.5176 | 0.0763 |
| (Iso)leucine* | 83 | 38 | 0.1047 | 0.0201 |
| Lysine* | 36 | 28 | 0.0216 | 0.0172 |
| Methionine | 9 | 9 | 0.1405 | 0.0667 |
| Phenylalanine* | 14 | 12 | 0.4645 | 0.0623 |
| Proline* | 24 | 21 | 0.0137 | 0.0135 |
| Serine | 33 | 13 | 0.0104 | 0.0108 |
| Threonine | 25 | 22 | 0.0149 | 0.0135 |
| Tryptophan* | 2 | 2 | 0.3232 | 0.0525 |
| Tyrosine* | 12 | 12 | 0.6785 | 0.0536 |
| Valine | 52 | 30 | 0.0128 | 0.0106 |

*Amino acids with immonium ions, described as diagnostically important. Leucine and Isoleucine were not considered to be distinguishable. The probabilities of immonium production were set to zero for alanine and glycine throughout the tuning process.

Copyright © 2004 John Wiley & Sons, Ltd. 

**Table 5.** Result of tuning on tuning dataset

| Correct sequence | Rank | Probability (%) | Top seq (untuned) | Rank | Probability (%) | Top seq (tuned) |
|---|---|---|---|---|---|---|
| SLGGEVFLDFTK | 15 | 0.27 | SLNEVFDLFTK | 4 | 9.43 | SLGGEVFDLFTK |
| LPLVGGHEGAGVVVGMGENVK | 374 | <0.01 | LPLVGGHEKRVVGMGENVK | 56 | <0.01 | LPLVGGHEGAGVVVGFAVNVK |
| ANGTTVLVGMPAGAK | >999 | <0.01 | KNTTVLVGMKPAK | 1 | 85.65 | ANGTTVLVGMPAGAK |
| TPEVDDEALEKFDK | 387 | <0.01 | TPEVQPPQASTHTDK | >999 | <0.01 | TPEVKMTNSVTDADK |
| VLVLDTDYKK | 371 | <0.01 | LVQEGQAYTGQ | >999 | <0.01 | LVQQGEAYASQ |
| TKLPAVFK | >999 | <0.01 | LLLYLLR | >999 | <0.01 | LRFRRR |
| TPEVDDEALEK | >999 | <0.01 | dPTTWPPEEK | >999 | <0.01 | TPEPPPMMAFK |
| SLAMAASDLSLLDAQSAPLR | >999 | <0.01 | SLAMAASDLSLLDALGGVLGR | 174 | <0.01 | SLTTAASDLSLLDAKSALPR |
| VLVLDTDYK | 253 | <0.01 | VLDELWYK | >999 | <0.01 | LVEDLWYK |
| TPEVDDEALEKFDK | >999 | <0.01 | TPSFEPASETEVVDK | >999 | <0.01 | SLGMNPSFGGEVPTDK |
| VAGTWYSLAMAASDLSLLDAQSAPLR | >999 | <0.01 | LPTGRSLLRRGKSDLSLNGLGLNNR | >999 | <0.01 | RWPLRRWSKRDLSLDGTQALPR |
| LDALNENK | >999 | <0.01 | QPTWEQK | >999 | <0.01 | QPTWEGAK |
| LSFNPTQLEEQCHL | >999 | <0.01 | FNRTHDKQGLPdGK | >999 | <0.01 | EFALPTRNHMTWR |
| SLAMAASDLSLLDAQSAPLR | >999 | <0.01 | LSMGRAMLSLLDAKSALPR | >999 | <0.01 | SLNWDLKLLSSDDSALPR |
| ALPMHLR | 2 | 48.75 | LAPMHLR | 1 | 52.68 | ALPMHLR |
| TPEVDDEALEKFDK | 1 | 31.71 | TPEVDDEALEKFDK | 1 | 78.13 | TPEVDDEALEKFDK |
| TPEVDDEALEKFDK | >999 | <0.01 | TPEVDDEAKLEFDK | 10 | 0.93 | TPEVDDEAKLEFDK |
| VLVLDTDYKK | 499 | <0.01 | VLVLdGLKYK | 45 | 0.16 | VLVLPAYKYK |
| SLAMAASDLSLLDAQSAPLR | >999 | <0.01 | SLAASLKADLLSDLSAADLR | 2 | 28.42 | SLAMAASLDSLLDAQSAPLR |
| TKLPAVFK | 2 | 22.69 | KTLPAVFK | 2 | 12.32 | KTLPAVFK |
| TPEVDDEALEK | 1 | 99.97 | TPEVDDEALEK | 1 | 99.96 | TPEVDDEALEK |
| VLVLDTDYK | 9 | 0.36 | VLVLPSFYK | 6 | 0.43 | VLVLPSFYK |
| VYVEELKPTPEGDLELLLQK | 62 | 0.02 | VYVEELQPTPWPFLLLLAGK | >999 | <0.01 | VYVEELQPTPWPFLLLLQK |
| VAGTWYSLAMAASDLSLLDAQSAPLR | >999 | <0.01 | LLLRYLRRRLLANYKRELYGK | >999 | <0.01 | DSDLdDDLQFRFLRGLKLLLLR |
| LDALNENK | 1 | 98.26 | LDALNENK | 1 | 61.80 | LDALNENK |
| VVGLSTLPELYEK | 1 | 38.08 | VVGLSTLPELYEK | 1 | 85.89 | VVGLSTLPELYEK |
| EKDLVGAVLK | 443 | <0.01 | GLSPMVKVLK | 378 | <0.01 | GLSPMVKVLK |
| EALDFFAR | 1 | 99.58 | EALDFFAR | 1 | 99.83 | EALDFFAR |
| YVVDTSK | >999 | <0.01 | YPTDTSK | 2 | 20.18 | YPTDTSK |
| GVLFYESHGK | 1 | 75.97 | GVLFYESHGK | 1 | 63.05 | GVLFYESHGK |
| ANELLLNVK | >999 | <0.01 | GQELLLNVK | 2 | 32.37 | NAELLLNVK |
| SLSLVGSYVGNR | 23 | 0.20 | SLSLVGSYVAAGK | 2 | 8.47 | LSSLVGSYVGNR |
| VLGLDGGEGKEELFR | >999 | <0.01 | VLGLDRGVVdVQFR | 133 | 0.04 | VLGLDGRVdVVQFR |
| VLGLDGGEGKEELFR | >999 | <0.01 | VLGLDMVLMLdALLG | >999 | <0.01 | VLGLDMVLEYLALGL |
| ATDGGAHGVLNVSVSEAALEASTR | >999 | <0.01 | ESAAVHVAAKGASVSEAALEASTR | >999 | <0.01 | KFSGMKLAASASVSEAALEKMR |
| LPLVGGHEGAGVVVGMGENVK | >999 | <0.01 | LPLVHGGEKGVVVGMGEGRK | >999 | <0.01 | LPLVGGHEKGVVVGMGEGRK |
| DLPVPKPK | 3 | 7.53 | DLPVKPPK | 3 | 15.90 | DLPVKPPK |
| LGDYAGLK | 89 | <0.01 | YATQLLK | 45 | <0.01 | AYTGALLK |
| ATDGGAHGVLNVSVSEAALEASTR | >999 | <0.01 | AGVGGMHGVLRGVKLLMAMKMR | >999 | <0.01 | VVdLHGVLHLFRLMAMMRK |

d, denotes Carboxyamidomethyl cysteine.

Copyright © 2004 John Wiley & Sons, Ltd.

**Table 6.** Result of tuning on validation dataset

| Correct sequence | Rank | Probability (%) | Top seq (untuned) | Rank | Probability (%) | Top seq (tuned) |
|---|---|---|---|---|---|---|
| GAAQNLLPASTGAAK | 327 | 0.00 | GAAQNLLQFALSH | 86 | 0.01 | AGAQNLLKFALSH |
| LVLNGHALTLFQER | 2 | 13.54 | LVLNGHALTLFAGER | 1 | 45.07 | LVLNGHALTLFQER |
| VVDLMVHMASKE | 9 | 0.06 | VVPMMVHMASKE | 76 | <0.01 | VVPMMVHMASEK |
| LVLNGHALTLFQERDPSNLK | 335 | <0.01 | LVLNGVGVHGNEWHFKGdLK | 0 | <0.01 | LVLRGMFELMEQLFHdLK |
| VLLSAPSADAPMFVMGVNHEK | 0 | <0.01 | VLLSAPSADAPMEGWWPRAGAGHDK | 0 | <0.01 | LVLSAPSADAPMFVYDYHEAASHK |
| YDDLKR | 1 | 94.63 | YDDLKR | 1 | 89.78 | YDDLKR |
| DAGAGLALNDHFVK | 0 | <0.01 | GEQVAALQNGESPK | 41 | 0.28 | ADGAGLALGGNAESPK |
| YDSTHGHFK | 1 | 91.70 | YDSTHGHFK | 1 | 94.71 | YDSTHGHFK |
| VLLSAPSADAPMFVMGVNHEK | 0 | <0.01 | APSDALQNGAQEDVFADAHGVT | 6 | 2.21 | VLLSAPASDAPMFVMGVNHEK |
| DAGAGLALNDHFVK | 3 | 10.33 | ADGAGLALNDHFVK | 3 | 10.00 | ADGAGLALNDHFVK |
| LVLNGHALTLFQERDPSNLK | 0 | <0.01 | LVLNGHALdLdLDLLLGVGGR | 0 | <0.01 | LVLNGHALQWGWQRLGKdK |
| VLHDNFGLVEGLMTTVHALTATQK | 0 | <0.01 | DLAVNNGVLVEGLMTTVHALFDLGP | 0 | <0.01 | EVGGNNLVLVEGLMTTVHALTHYK |
| LEKPAKYDDLK | 0 | <0.01 | LEKTGdKELLK | 0 | <0.01 | YFVPPNTQLLK |
| VVDLMVHMASKE | 2 | 38.61 | VVPMMVHMASKE | 1 | 52.76 | VVDLMVHMASKE |
| APLLAVTR | 1 | 100.00 | APLLAVTR | 1 | 100.00 | APLLAVTR |
| VNLAMNVGK | 1 | 99.61 | VNLAMNVGK | 1 | 99.68 | VNLAMNVGK |
| LDLDSAPLTAR | 1 | 97.09 | LDLDSAPLTAR | 1 | 87.50 | LDLDSAPLTAR |
| RFDELLEASDGLMVAR | >999 | <0.01 | FDRELLEASDGLMVAR | 19 | 0.05 | QNSAFLLEASDGLMVAR |
| GVNLPGAAVDLPAVSEK | >999 | <0.01 | GVNLPQAVDLFGLFF | >999 | <0.01 | GVNLPQAVDLLWRR |
| GDLGLELPAEK | 6 | 0.11 | GLDGLELPAEK | 7 | 0.17 | GLDGLELPAEK |
| LTLDNAYMEK | 1 | 96.89 | LTLDNAYMEK | 1 | 99.86 | LTLDNAYMEK |
| MQHLLAR | 2 | 19.05 | MKHLLAR | 2 | 6.92 | MKHLLAR |
| VYVDDGLLSLQVK | 5 | 1.32 | VYVDDAVLSLQVK | 2 | 10.98 | VYVDPGMLSLQVK |
| KLFEELAR | >999 | <0.01 | KLFNGSLAR | >999 | <0.01 | KLFNELNK |
| GSGTAEVELK | 2 | 27.34 | GSTGAEVELK | 3 | 12.19 | GSGTAPMELK |
| LFEELAR | 1 | 94.78 | LFEELAR | 1 | 92.95 | LFEELAR |
| RPVAVALDTK | 1 | 97.94 | RPVAVALDTK | 1 | 99.96 | RPVAVALDTK |
| FDELLEASDGLMVAR | 49 | 0.15 | DFELLEAMAGLMGLR | 16 | 1.26 | DFELLEAMAAVMGLR |
| GDYPLEAVR | 1 | 69.76 | GDYPLEAVR | 1 | 56.29 | GDYPLEAVR |
| FGVEQDVDMVFASF | >999 | <0.01 | VGDQGDFQSSVFASF | >999 | <0.01 | GVTNPTMFGGGTFASF |
| MNFSHGTHEYHAETLK | >999 | <0.01 | MNEHVDADQTFAQMHK | 505 | <0.01 | MNMVLHDAGAHGDQFMK |

Copyright © 2004 John Wiley & Sons, Ltd.

production, and confirmation for others in the original set. However, the results are not markedly different and we conclude that a single set of reasonable parameters will suffice to give good results over a range of instruments and the details of their configurations.

We have put in place a flexible, automated scheme for tuning the prior used in the calculation of the likelihood Pr(Spectrum | Sequence), used in both *de novo* sequencing and databank searching applications, to which new data can easily be added and the fragmentation characteristics observed from new instruments and experiments accommodated.

### Acknowledgements

KR would like to thank the organizers of the MIPNETS meeting held in Liverpool, UK, in June 2003 for providing the opportunity to present and discuss this work.

### References

1. Hastings WK. 1970. *Biometrika* **57**: 97–109.
2. Kirkpatrick S, Gelatt CD, Vecchi MP. 1983. *Science* **220**: 671–68.
3. Morris HR, Paxton T, Dell A *et al*. 1996. *Rapid Commun Mass Spectrom* **10**: 889–896.
4. Metropolis N, Rosenbluth AW, Rosenbluth MN *et al*. 1953. *J Chem Phys* **21**: 1087–1092.
5. Papayannopoulos IA. 1995. *Mass Spectrom Rev* **14**(1): 49–73.
6. Skilling J. 2000. US Patent No. 6489608.
7. Skilling J. 2000. US Patent No. 6489121.
8. Skilling J. 1998. *J Microsc* **190**: 28–36.

Copyright © 2004 John Wiley & Sons, Ltd.
